# How Light Modulates the Growth of *Cyanidioschyzon merolae* Cells by Changing the Function of Phycobilisomes

**DOI:** 10.3390/cells12111480

**Published:** 2023-05-26

**Authors:** Tomasz Krupnik, Maksymilian Zienkiewicz, Wioleta Wasilewska-Dębowska, Anna Drożak, Kinga Kania

**Affiliations:** Department of Molecular Plant Physiology, Institute of Environmental Biology, Faculty of Biology, University of Warsaw, Miecznikowa 1, 02096 Warsaw, Poland

**Keywords:** allophycocyanin, chlorophyll *a*, fluorescence, *Cyanidioschyzon merolae*, growth rate, respiration rate, photosynthesis rate, energetic status, light quality, light intensity, phycobilisomes, phycocyanin

## Abstract

The aim of this study was to examine how light intensity and quality affect the photosynthetic apparatus of *Cyanidioschyzon merolae* cells by modulating the structure and function of phycobilisomes. Cells were grown in equal amounts of white, blue, red, and yellow light of low (LL) and high (HL) intensity. Biochemical characterization, fluorescence emission, and oxygen exchange were used to investigate selected cellular physiological parameters. It was found that the allophycocyanin content was sensitive only to light intensity, whereas the phycocynin content was also sensitive to light quality. Furthermore, the concentration of the PSI core protein was not affected by the intensity or quality of the growth light, but the concentration of the PSII core D1 protein was. Finally, the amount of ATP and ADP was lower in HL than LL. In our opinion, both light intensity and quality are main factors that play an important regulatory role in acclimatization/adaptation of *C. merolae* to environmental changes, and this is achieved by balancing the amounts of thylakoid membrane and phycobilisome proteins, the energy level, and the photosynthetic and respiratory activity. This understanding contributes to the development of a mix of cultivation techniques and genetic changes for a future large-scale synthesis of desirable biomolecules.

## 1. Introduction

*Cyanidioschyzon merolae* is a species of eukaryotic extremophilic red algae that thrives in low pH environments (approximately 0.2 to 4) and moderately high temperatures (40–56 °C) [1]. It is typically characterized by a small size (1.5 × 3.5 μm) and extremely simple anatomy, with a single chloroplast, mitochondrion, and nucleus. Although the nuclear, plastid, and mitochondrial genomes have been sequenced [2,3,4], little research has been done on the physiology of light adaptation of the photosynthetic apparatus to various intensities and qualities. *C. merolae* contains only chlorophyll *a*, which absorbs blue and red light most efficiently. In our previous work [5], we observed that its photosynthesis and CO_2_ absorption reached approximately half the efficiency in both red and blue light compared to white light of the same intensity. In addition, this organism expresses secondary light-harvesting antennae (PBSs), capable of absorption of light in the yellow part of the spectrum. This mode of light harvesting is common among cyanobacteria and red algae. Phycobilisomal antennae are large hemidiscoidal or hemispherical structures that protrude from the chloroplast thylakoid membrane into the stroma [6]. Their superstructure is composed of two main classes of proteins: phycocyanin (R-PC) with maximum absorption at 620 nm and allophycocyanin (APC) with maximum absorption at 650 nm [7] containing two or three chemically bound chromophore molecules involved in light harvesting and transfer of energy to photosynthetic reaction centers [8]. Energy transfer is associated with an increase in the wavelength of emission from a decoupled PBS component and a decrease in the lifetime of excited states [9]. PBS undergoes structural reorganization as an effect of adaptation to the light intensity and its specific wavelength. Cells grown in monochromatic LED light were capable of reconstructing the structure of phycobilisomes [10], resulting in defective energy transfer from APC to chlorophyll (Chl). This effect was observed in all monochromatically grown cells of *C. merolae,* except the control grown under white light. The higher relative content of R-PC was coupled with a more efficient transfer of energy from R-PC to APC. The APC had the lowest efficiency of energy transfer, suggesting that it might constitute a regulatory element in light acclimation. These differences in fine-tuning of the photosynthetic apparatus of *C. merolae* in various light regimes might be particularly useful in biotechnological applications, especially those requiring the production of deleterious or toxic compounds or proteins. It could be possible to produce a culture of high density in yellow light and then induce production of high-value products with blue light irradiation. The growing availability of transformation protocols for *C. merolae* [11] and other algae [12] allows for the reengineering of their metabolism in a variety of ways for appropriate biotechnological production. Further analysis of genetic and physiological interactions is needed to identify the appropriate transformation techniques, gene promoters, or cellular compartments for expression [13,14] and for optimizing the application of algae as bio factories.

In this work, we intended to elucidate the influence of light quality (wavelength) and quantity (intensity) on the photosynthetic capacity of *C. merolae* with special attention to the remodeling of the phycobilisomal antennae. We grew *C. merolae* cultures in low light intensity (LL) and high light intensity, provided by white (W), blue (B), yellow (Y), and red (R) color LEDs. Yellow and red light did not induce faster growth rates than the white light control. We propose that the reason for this behavior is the complicated interplay of synthesis and degradation of the protein components of PBS, their association and dissociation from photosystems, and the modulation of photosystem II synthesis. Our data provide strong evidence that light quality and low light intensity are the main regulators of cell function by modulating PBS.

## 2. Materials and Methods

### 2.1. Cell Cultures

*Cyanidioschyzon merolae*, 10D strain (NIES-1332, Unialgal, Clonal and Nonaxenic), was obtained from the Microbial Culture Collection (mcc.nies.go.jp, Tsukuba, Japan) and was used throughout this study. Cells were grown in MA2 liquid medium [15] in a glass vessel shaken at 100 rpm under continuous white light illumination (100 μmol photons m^−2^ s^−1^) at 40 °C.

*Escherichia coli*, strain DH5α (genotype: F− Φ80lacZΔM15 Δ (lacZYA-argF) U169 recA1 endA1 hsdR17 (rK−, mK+) phoA supE44 λ− thi-1 gyrA96 relA1), were used for the construction of transformation vectors [16]. Bacterial cells were cultured in liquid LB medium (1% Bacto tryptone, 0.5% yeast extract, 1% NaCl at 37 °C) or in Petri dishes with LB medium solidified by the addition of 1% agar. For the selection of transformed cells, the medium was supplemented with ampicillin (50 µgmL^−1^) (Roth, Germany).

### 2.2. Cyanidioschyzon merolae Growth Rate Study

Cells were grown in a multicultivator (PSI, Czechia), and growth curves were recorded by acquiring OD values at wavelengths of 680 nm and 720 nm at 30 min intervals. A volume of approximately 80 mL of cells at the initial OD_720_ = 0.01 was illuminated with the light intensities of 25, 50, 100, and 250 μmol photon m^−2^ s^−1^ of integrated LED light for 168 h (7 days) in a continuous flow of sterile air 0.5 Lmin^−1^ and 40 °C. For this study, four wavelengths were selected, white (control), blue (450 nm) yellow (615 nm), and red (660 nm). For rate comparison purposes, the growth rates were calculated in the linear area of highest growth in light intensities of 25, 50, 100, and 250 μmol photon m^−2^ s^−1^ of white, blue, yellow, and red light, and subsequently, averages of at least three measurements are presented.

### 2.3. SDS-PAGE and Immunoblotting Analysis

Cells, grown under various conditions, were harvested by spinning for 5 min at 2000× *g* and resuspended in the buffer (20 mM HEPES-NaOH (pH 7.6), 5 mM EDTA, and 330 mM sucrose). Chlorophyll concentration was quantified spectrophotometrically (UV-1800 Shimadzu, Japan) by measuring absorption at 663 nm of chlorophyll *a*, extracted with 80% (*v*/*v*) acetone. Numerical values were derived from the Beer–Lambert equation, the extinction coefficient ε = 85.95 (L g^–1^ cm^–1^) [17], and the dilution factor. In addition, fresh samples of cells were counted in the Neubauer fluorescent chamber (Marienfeld, Germany). For electrophoresis cell samples, in a number of 10^7^ cells, measures were taken, pelleted, and solubilized in denaturing buffer (0.25 M Tris-HCl (pH 6.8), 0.4% (*w*/*v*) SDS, 10 M urea, 2% (*v*/*v*) 2-mercaptoethanol, and 20% (*v*/*v*) glycerol). Proteins were separated on 12% gels by the Laemmli-type SDS-PAGE method [18]. The gel wells were loaded with 10 µL of samples (0.2–1 μg of Chl *a*) and run at constant voltage (75 V). Following electrophoresis, polypeptides were electrotransferred onto the PVDF membrane [19] and probed with rabbit anti-APC (specific to allophycocyanin α and β), rabbit anti-R-PC (specific to R-phycocyanin), anti-D1 (specific to D1 subunit of PSII), or PsaA (PSI-A core protein of photosystem I) antibodies (Agrisera, Sweden). Bands specifically binding the probe were visualized by the enhanced chemiluminescence method according to standard procedures using the ChemiDoc System (Bio-Rad Laboratories, Inc., Berkley, CA, USA). The DNA sequences of standard proteins of (PsaA, R-PC, and APC) were amplified by PCR from genomic DNA of *C. merolae* and cloned into a pBAD24 vector with C-terminal HisTag and purified on the HisTrap column by HPLC (the list of primers is found in Table S1). The PsbA standard was obtained directly from the purification of PSII from *C. merolae* [20]. The fractions of greatest purity were collected and pooled, and the concentration was established by SDS-PAGE electrophoresis with BSA standard. The core proteins were probed with antibodies. The intensity of the relevant bands was measured and interpolated with the known concentration of standard proteins.

### 2.4. Production and Purification of Protein Standards

For the semi-quantitative determination of the supercomplex components, relevant fragments (containing the epitope as described by the antibody producer or are most likely to contain it) of their genes were cloned into the expression vector. The extraction of genomic DNA from *C. merolae* was carried out by lysis of 10^8^ cells in 0.75 mL of CTAB buffer (CTAB 20.00 g/L (2% *w*/*v*) EDTA·Na_2_·2H_2_O 7.44 g/L (20 mM) NaCl 81.82 g/L (1.4 M) Tris 12.11 g/L (100 mM)) and the subsequent addition of 0.75 mL of chloroform/isoamyl alcohol (24:1). The mixture was vortexed for 5 min and centrifuged for 15 min at 4 °C at maximum speed. The upper (aqueous) fraction was transferred to a fresh 2 mL tube in a volume of 360 µL with 1440 µL ice-cold ethanol (Sigma, Darmstadt, Germany) and 200 µL of 10 mM ammonium acetate. The samples were kept at −20 °C for 30 min and then centrifuged for 30 min. The pelleted DNA was washed twice with ice-cold ethanol and allowed to dry. The pellets were dissolved in sterile water, and the concentration of DNA was measured by nanodrop (Thermo Fisher Scientific Inc., Waltham, MA, USA). The genes of phycocyanin (*R-PC*, Q85G43), allophycocyanin (*apcA*, Q85FW7), D1 (*psbA*, Q85G59), and PSI (*psaA*, Q85FY7) were amplified by PCR with primers, carrying relevant restriction sites (NcoI and XbaI, Table S1). The sites were selected to use the N-terminal 6xHisTag label, present in the pBAD plasmid. Plasmids and PCR products were digested with selected enzymes (Thermo Fisher Scientific Inc., Waltham, MA, USA), separated on 1% agarose gel, excised, and recloned with the ligase T4 reaction (Thermo Fisher Scientific Inc., Waltham, MA, USA). The ligation products were transformed into rubidium-competent DH5α *E. coli* cells and spread on 1% agarose 100 µg mL^−1^ ampicillin selective Petri plates. Up to three single colonies were selected from each plate, and isolated plasmids were sequenced. The correct plasmids were transformed into rubidium-competent DH5α *E. coli* cells and cultured overnight in 10 mL of LB growth medium (Carl Roth GmbH + Co. KG, Karlsruhe Germany) in a 50 mL flask in the presence of 100 µg mL^−1^ ampicillin. The following day, 10 mL of cell suspension was transferred to 1 L of fresh LB medium in 5 L culture flasks. The growth rate was monitored every hour until the OD value of the OD at 660 nm reached 0.4. At this point, protein expression was induced with 1 g of arabinose (working solution 1 mg mL^−1^) and incubated for one hour. Subsequently, the cells were cooled on ice for 30 min and spun at 8000 rpm for 10 min. Cell pellets were resuspended in 10 mL of 50 mM NaPO_4_ buffer (pH 7.0; 57.7/42.3 Na_2_HPO_4_/NaH_2_PO_4_) and spun down again to wash out any remaining LB. The final suspension volume of 5 mL was sonicated (0.25/0.75 s on/off for 15 min) to achieve cell lysis, and then 1% SDS was added and overnight incubation was carried out to solubilize any possible inclusion bodies.

The solubilized cells were diluted in 15 mL of carrier buffer (20 mM sodium phosphate, 0.5 M NaCl, 20 mM imidazole; pH 8.0) and ultracentrifuged at 40,000 rpm for 30 min, and then the supernatant was filtered and applied on the preparative HPLC (Sykam GmbH, Eresing, Germany) with a 1 mL Ni-NTA resin column (HisTrap, Merck KGaA, Darmstadt, Germany). The system was equilibrated with carrier buffer (20 mM sodium phosphate, 0.02 M NaCl, 20 mM imidazole; pH 8.0) at 1 mlmin^−1^ flow for 5 min, followed by 5 mL sample injection. The column was washed with buffer A for 5 min and after a 10 min gradient (0–80%) of elution buffer B (20 mM sodium phosphate, 0.5 M NaCl, 500 mM imidazole; pH 7.0) was applied. The peak fractions were collected and analyzed on SDS-PAGE. Several of the purest fractions were pooled and concentrated in centrifuge concentrators VivaSpin 6, 10,000 MWCO (Sartorius AG, Göttingen, Germany), and the concentration was approximated with standard BSA (Sigma-Aldrich, Germany) standard protein on Coomassie Brilliant Blue G250-stained SDS-PAGE gel by densitometric measurements.

### 2.5. Determination of ATP and ADP Content in Whole Cells

Cell samples were harvested from fresh cell cultures grown to OD_720_ = 0.2 in MA2 medium. Cells were centrifuged at 16,900× *g* at 4 °C for 5 min in the dark and immediately ground in liquid nitrogen. The powder obtained was treated with 10% (*v*/*v*) HClO_4_ and left for 5 min on ice. The ice-cooled samples were centrifuged at 10,000× *g* for 2 min, and the aliquots of the supernatants were brought to pH 7.0 by adding 1 M triethanolamine in 5 M KOH. After 30 min on ice, the precipitated KClO_4_ was pelleted (10,000× *g* for 2 min), and the adenylate content was measured in the supernatants. ATP was determined by the firefly luciferase method [21]. ADP was converted to ATP by pyruvate kinase (Boehringer, Mannheim, Germany) and determined as described above. Each measurement was calibrated with the addition of the ATP standard. The measurements were repeated at least three times in three to four separate experiments.

### 2.6. Starch Concentration Measurements

Starch was measured with the spectrophotometric method, on the reaction of starch with the Lugol solution. Cells were harvested in equal numbers (50 × 10^8^ cells) under all experimental conditions. Cells were spun for 10 min and at 4 °C in a tabletop centrifuge (5810 R, Eppendorf, Hamburg, Germany) at 5000 rpm. The pellet was resuspended in 10 mL of ethanol (Chempur, Piekary Śląskie, Poland) and vortexed for 5 min. The extracted chlorophyll was removed by centrifuging the cellular debris at 5000 rpm and 4 °C for 10 min. The pellet was resuspended in 1 mL of MQ water and heated to 95 °C for 15 min. The debris was spun down at 5000 rpm and 20 °C for 1 min. Subsequently, 1 mL of supernatant was mixed with 1 µL of 10% Lugol solution and thoroughly mixed until a bluish color appeared. The absorbance was measured in a 1 mL cuvette in a spectrophotometer (UV-1800 Shimadzu Corporation, Nishinokyo Kuwabara-cho, Japan) at 525 nm. The standard line was prepared by dissolving 100 mg of starch standard (POCH, Avantor Performance Materials Poland SA, Gliwice, Poland) in 10 mL of water by boiling for 10 s in a microwave oven. The required concentrations were prepared from the stock solutions (5 standards, from 0.01 to 0.25 mg/mL) and mixed with 1 µL of 10% Lugol solution.

### 2.7. Determination of Chlorophyll a Concentration

Analytical high pressure liquid chromatography (HPLC), using the Shimadzu Prominence System with PDA detector (Shimadzu Corporation, Nishinokyo Kuwabara-cho, Kyoto, Japan), was performed according to a modified method of Krupnik et al. [20] using a maximum flow rate of 1 mL min^−1^ and a Bionacom 3000 C18 column (Bionacom LTD, Coventry, UK). Pigments were extracted from 10^8^ cells (harvested from fresh cultures at OD 668 = 0.2) with 1 mL of 80% acetone. Cell suspension volumes were not greater than one-quarter of the extraction mixture. Cell debris was removed by 10 min centrifugation at 4 °C in a tabletop microcentrifuge. The extract was concentrated in a SpeedVac (Concentrator Plus, Eppendorf, Hamburg, Germany) in a 30 °C centrifuge until it dried out. The samples were dissolved in 50 µL of acetonitrile/triethylamine (99.9:0.1, *v*/*v*) and loaded onto the C18 column that was previously equilibrated with phase A (acetonitrile: methanol, 6:2 *v*/*v*). The pigments were separated with a linear gradient of 0–75% of phase B (ethyl acetate), starting at the 10th minute of the run. The concentration was calculated as the area under the corresponding pigment peak. The molar extinction coefficients at the wavelength λ = 436 nm in acetonitrile were the following: 91.7 mM^−1^cm^−1^ for Chl *a* and 28.5 mM^−1^ cm^−1^ for pheophytin [22]. Rare incidences of chlorophyll degradation resulted in the appearance of pheophytin, in which a molar contribution was added to the moiety of chlorophyll.

### 2.8. Oxygen Exchange Activity of Cells (Photosynthesis and Respiration Rate)

The functional activities of cell suspension (0.1–0.5 × 10^8^ cells) from all growth light conditions (white (W), yellow (Y), blue (B), and red (R) of low and high light intensity) were measured using an oxygen Clark-type electrode (Hansatech, King’s Lynn, UK) with W, B, Y, and R lights. Measurements were performed at 30 °C in 1 mL of fresh MA2 liquid medium [20], supplemented with 10 µL of 100 mM NaHCO_3_ (Chempur, Piekary Śląskie, Poland) as an exogenous source of CO_2_. Samples were illuminated with the standard white light intensity of 25, 100, 500, and 1500 µmol photons m^−2^ s^−1^ using a KL 2500 LCD white light source (Schott AG, Mainz, Germany) or a monochromatic LED of blue (450 nm), yellow (600 nm), and red (650 nm) light (Brilliance LED LLC, Carefree, AZ, USA). Initial activities were calculated from the photosynthetic oxygen evolution curve and the respiration curve, acquired three times for every condition of light intensity and quality. Cell counts were measured with the Neubauer chamber as an average of at least 10 counts. Following a 1 min incubation, changes in oxygen concentration were recorded for 3 to 5 min. Subsequently, the light was turned off, and the dark respiration was measured for an additional 3 to 5 min. The initial rates of oxygen exchange were calculated from angulation of the line, tangent to the light curve, measured in the light range between 25 and 1500 μmol photons m^2^ s^−1^ of white light (Schott AG, Mainz, Germany) as well as blue, yellow, and red lights from LED sources (Brilliance LED LLC, Carefree, AZ, USA).

### 2.9. 77 K Fluorescence

The low temperature (77 K) fluorescence spectra were acquired using an Agilent Technologies Cary Eclipse Fluorescence Spectrophotometer with a cryostat (Oxford Optistat DN2, Oxford Instruments plc, Abingdon, UK). Cell suspensions were mixed with 1 mL of 50% glycerol, placed in a fluorescence cuvette, and frozen in liquid nitrogen. The frozen cuvette was placed in the cryostat sample holder and within the optical path of the fluorimeter. The sample was left to reach a precise temperature of 77.5 K, and spectra were acquired within the 600–800 nm fluorescence range with a 580 nm excitation beam.

## 3. Results

### 3.1. Cell Growth Rates

**Figure 1:**
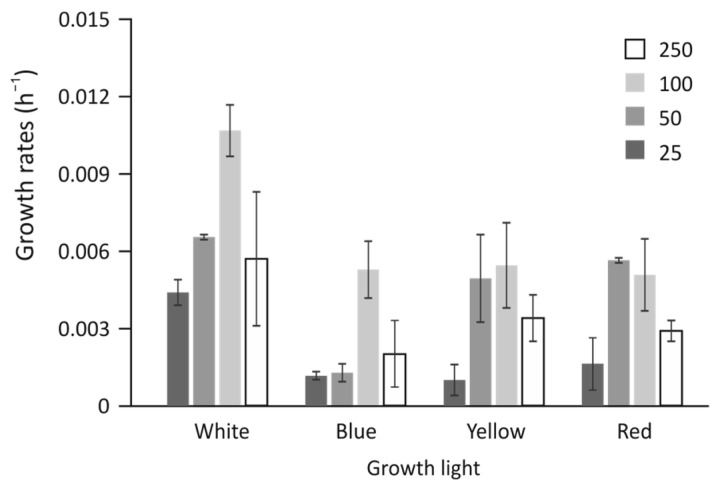
Cellular growth dynamics. Cells were grown in white, blue, yellow, and red light at 25, 50, 100, and 250 µmol photons m^−2^ s^−1^ (legend shown in the figure inset), and growth curves were recorded by monitoring the OD_720_. Average values of at least three growth rate measurements are presented.

*Cyanidioschyzon merolae* cells were grown at four selected light intensities (25, 50, 100, and 250 μmol photons m^−2^ s^−1^) in a multicultivator (PSI, Czechia) at four wavelengths: blue (B, 450 nm), yellow (Y, 615 nm), red (R, 660 nm), and white (W, control) light at 40 °C with constant aeration of 0.5 dm^3^ min^−1^. The growth curves were automatically recorded at 680 and 720 nm. Growth rates were calculated from an average of at least three measurements of a 7-day culture at OD 720 nm for comparison purposes. It was concluded that growth strongly depends on both the intensity of light and the quality of growth light (Figure 1). The fastest and optimal growth was observed in the W at 100 μmol photons m^−2^ s^−1^. The higher light intensity (250 μmol photons m^−2^ s^−1^) resulted in ≈50% reduction in growth rate. The effect was similar in the case of a lower light intensity (50 μmol photons m^−2^ s^−1^), with a further reduction, to ≈30% in 25 μmol photons m^−2^ s^−1^. All monochromatic lights caused a reduction in the growth rate, down to at least 50% of the white control. The B light exhibited possibly the greatest reduction in the rate of growth at all light intensities compared with white light of the relevant intensity. The least affected was the culture grown at 100 μmol photons m^−2^ s^−1^ of B, where it achieved ≈50% white light control similar to other monochromatic lights of 100 μmol photons m^−2^ s^−1^. Both R and Y light exhibited similar growth characteristics in all light intensities, with a 70% reduction in 25 μmol photons m^−2^ s^−1^. It was observed that the Y and R light of 50 and 100 μmol photons m^−2^ s^−1^ had almost identical effects on the growth rate and spanned the apparent optimum of growth.

### 3.2. Level of PsaA, PsbA, APC, and R-PC Proteins

Cells were grown in white light and three selected light regimes (blue, yellow, and red) of continuous light at two intensities: 25 (LL) and 100 (HL) μmol photons m^−1^ s^−1^ in the multicultivator (PSI (Photon Systems Instruments), spol. s r.o., Drásov, Czechia). The harvested cells were counted in a Neubauer chamber, and 10^6^ cells were lysed and applied to SDS-PAGE gel for electrophoresis with a subsequent transfer to a PVDF membrane. The samples, together with the recombinant standard protein, as well as the size marker, were loaded onto the gel.

**Figure 2:**
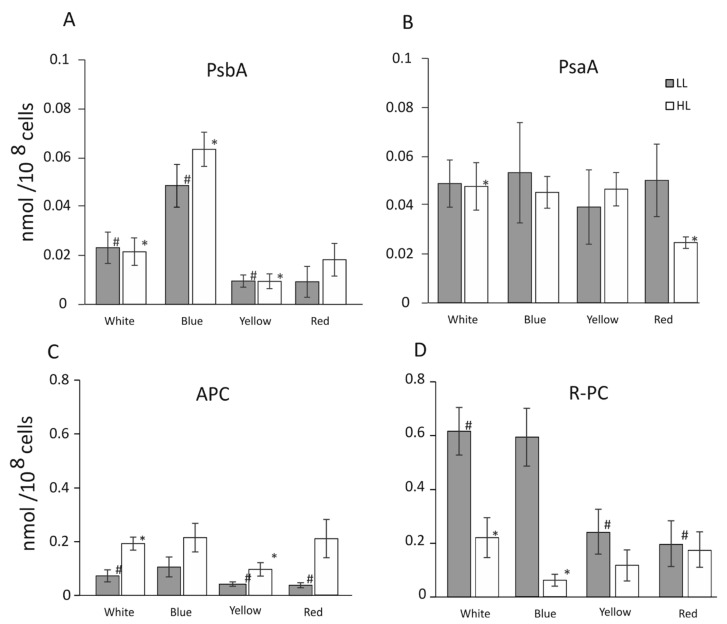
Effect of W, B, Y, and R light of low intensity (gray bars, LL) and high intensity (white bars, HL) (25 and 100 µmol photons m^−2^ s^−1^) on the levels of proteins PsbA (**A**), PsaA (**B**), APC (**C**), and R-PC (**D**) in *C. merolae* cells. Immunoblot densitometric measurements were repeated at least 3 times. The *t*-test (T.TEST, Excel, Microsoft) returned statistically significant results (*p* < 0.05) that were indicated by * and # and applied to the high and low light controls, respectively.

The concentration of the PSII D1 core protein (PsbA) was observed to not change significantly upon the increase in light intensity, yet it was highly regulated by light quality (Figure 2A). B-HL and B-LL appeared to highly upregulate the expression of PSII core proteins, allowing them to increase its concentration three times, up to 0.06 nmol 10^−8^ cells, compared with 0.02 nmol 10^−8^ cells in the control W-HL or W-LL. Yellow light reduced the abundance of PsbA by ≈50% in both light intensities, down to 0.01 nmol 10^−8^ cells compared to white control light. Red light was able to reduce the abundance of the D1 protein to ≈50% of the white control, but only at low light intensity (Figure 2A). In R-HL, the level of D1 protein was unchanged, compared with the white control. Unlike the PSII protein of PSI, the PSI core protein (PsaA) was not markedly susceptible to light intensity or quality, except for R-HL, which reduced the abundance of PsaA by ≈50% (Figure 2B). It was observed that the abundance of allophycocyanin (APC) was modulated first by the light intensity and to a lesser extent by a specific wavelength. Its abundance in B light was essentially identical to the W control and was reduced in Y and R light by ≈50% in low light alone (Figure 2C). The abundance of APC was roughly doubled in HL, regardless of the wavelength of the growth light. The concentration of phycocyanin (R-PC) was highly dependent on both the intensity and quality (Figure 2D). The W-LL and B-LL caused intensive induction of R-PC expression at the level of ≈0.6 nmol 10^−8^ cells and reduction to ≈0.3 nmol 10^−8^ cells in Y and R light. The high light intensity had a very distinctive inhibitory effect on the level of the R-PC protein in W light, where it was reduced to ≈30% of its W-LL value and reached 0.2 (nmol 10^−8^ cells). B-HL had a much more pronounced inhibitory effect, leading to a tenfold decrease in R-PC (0.05 nmol 10^−8^ cells). Y-HL had a lower inhibitory effect, allowing for ≈50% reduction of R-PC to approximately 0.1 (nmol 10^−8^ cells). The R growth light had almost no influence on the amount of R-PC—the level was similar (0.2 nmol 10^−8^) in cells grown in the HL and LL.

### 3.3. Initial Photosynthesis and Respiration of C. merolae Cells

**Figure 3:**
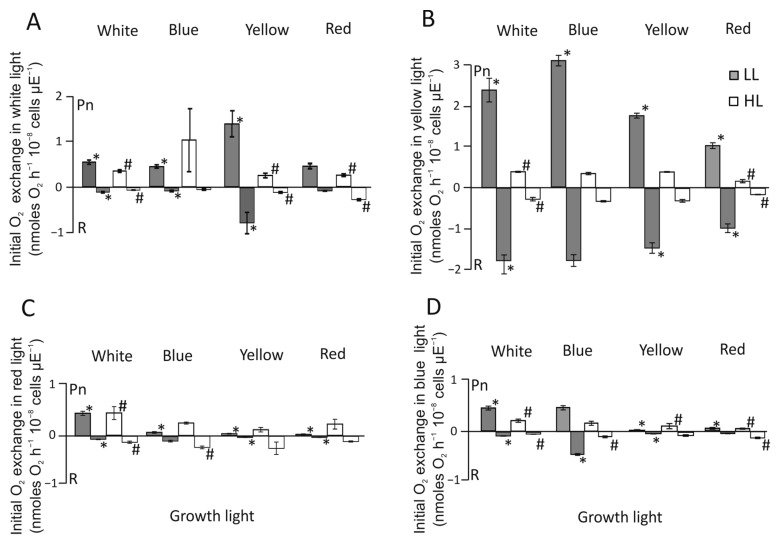
The initial photosynthesis (Pn) and respiration (R) rates of *C. merolae* cells measured in white (**A**), yellow (**B**), red (**C**), and blue (**D**) light at LL (gray bars) and HL (white bars) (25 and 100 µmol photons m^−2^ s^−1^). Oxygen exchange was measured at all light intensities for cells under different light growth conditions. Respiration rates were measured in darkness 5 min after the onset of photosynthesis. Results are the mean ± SD of at least three independent experiments. The *t*-test (T.TEST, Excel, Microsoft) returned statistically significant results (*p* < 0.05) for Pn and R, separately, which were denoted with * and # and applied to the high and low white light controls, respectively.

The initial oxygen-evolving activities of *C. merolae* cells, grown in the W, Y, R, and B of both light intensities (LL and HL), were recorded using a Clark-type oxygen electrode (Hansatech, UK). It was observed that Y-LL (25 μmol photons m^−2^ s^−1^) was able to induce the highest initial rate of oxygen evolution, when measured in white light (Figure 3A), reaching 1.3 nmol O_2_ h^−1^ 10^−8^ cells μE^−1^ of the initial rate, while for cells growing in Y-HL, the initial photosynthesis rate of photosynthesis was only 0.3 nmol O_2_ h^−1^ 10^−8^ cells μE^−1^, while the control conditions (W cells, Figure 3A) exhibited only 0.5 and 0.4 nmoles O_2_ h^−1^ 10^−8^ cells μE^−1^ for LL and HL, respectively. B-HL also showed high activity in the W measure light, reaching 1 nmoles of O_2_ h^−1^ 10^−8^ cells μE^−1^. In almost all cases, the initial rates of dark respiration values reached a similar low value of 0.1 nmol O_2_ h^−1^ 10^−8^ cells μE^−1^, with the notable exception of B-LL and R-HL. In all growth conditions, the initial photosynthesis rates, measured in yellow light (Figure 3B), showed the strongest induction of oxygen evolution. Cells grown in W-LL and B-LL exhibited 5–6 times higher initial oxygen evolution measured in yellow light (Figure 3B) compared to the respective white light (Figure 3A). Cells grown in yellow light had an almost identical initial rate of photosynthesis during measurements in white and yellow measure light, respectively (Figure 3A,B). The initial rates of oxygen-evolving activity of cells measured in red (Figure 3C) and blue (Figure 3D) light were never higher in either light intensity than in the white control and generally in the range of 20–50% of its value (Figure 3C,D). The highest values of 0.5 nmol O_2_ h^−1^ 10^−8^ cells μE^−1^ were observed for W-LL during measurement in light R and B (Figure 3C,D, respectively). Interestingly, cells grown in B-LL were able to produce a high rate (0.5 nmol O_2_ h^−1^ 10^−8^ cells μE^−1^) of initial photosynthesis (Figure 3D) measured in B, comparable to the same cells grown in W (Figure 3D).

The relationship between the initial rates of photosynthesis and respiration appeared to be proportional. The initial rates of respiration always followed the values of the initial rates of oxygen evolution during photosynthesis, reaching high values only when the same cells reached high oxygen evolution. Values of the initial rates of photosynthesis and respiration of *C. merolae* cells measured in YL showed a significant stimulation of both processes, regardless of quality of the growth light, reaching between ≈1.5 and up to ≈3.5 nmoles O_2_ h^−1^ 10^−8^ cells μE^−1^ (Figure 3B). The exception was observed for cells grown in R-LL, where both initial rates of oxygen evolution and respiration reached ≈1 nmoles O_2_ h^−1^ 10^−8^ cells μE^−1^ (Figure 3B). Interestingly, cells grown in HL showed very low initial rates of oxygen evolution and respiration at ≈0.5 nmoles O_2_ h^−1^ 10^−8^ cells μE^−1^, similar to the white control. Both respiration and oxygen evolution initial rates, measured in R and B (Figure 3C,D, respectively), were similar in both light regimes and had the highest values in W-LL, reaching ≈0.5 nmoles O_2_ h^−1^ 10^−8^ cells μE^−1^ for oxygen evolution and ≈0.2 nmoles O_2_ h^−1^ 10^−8^ cells μE^−1^ in respiration. The notable exception was the respiration rate of B-LL cells, measured in R light (Figure 3D), reaching ≈0.5 nmoles O_2_ h^−1^ 10^−8^ cells μE^−1^.

### 3.4. The Concentration of Chlorophyll a in C. merolae Cells

**Figure 4:**
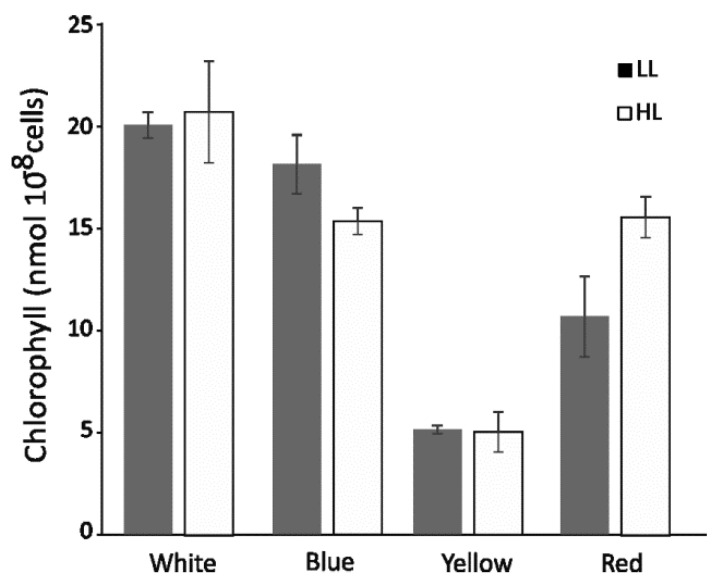
Chlorophyll *a* concentration in *C. merolae* cells growing in white, blue, yellow, and red light. Low (LL) and high (HL) intensity of growth light is marked with gray and white bars, respectively. Results are mean ± SD of at least three independent experiments.

The chlorophyll *a* concentration was assessed by HPLC and interpolated with the standard curve. Approximately 10^8^ cells from all growth conditions were collected, and chlorophyll *a* was extracted with 1 mL of 80% (*v*/*v*) acetone and analyzed. The chlorophyll *a* at its highest value was approximately 20 nmol 10^−8^ cells for cells grown in white light (Figure 4).

The level of chlorophyll *a* was insensitive to either intensity of W and Y growth light, while it decreased by 10% in B-HL and increased by 20% in R-HL (Figure 4) when compared to the low light control of the same color. The chlorophyll *a* was highly sensitive to light quality of light during growth. In blue light, Chl *a* decreased by 10% in low light and about 20% in high light as compared to the white-light control. Yellow light caused a significant reduction of chlorophyll concentration by ≈75%, regardless of the growth light intensity. Red light-induced ≈40% reduction of chlorophyll *a* in the low light but only 20% in the high light as compared to the white control.

### 3.5. Fluorescence of Free Phycobilisomes

Cells of *C. merolae*, grown under all previously described conditions, were diluted to identical OD_720_ = 0.2 as described in the Materials and Methods section. Immediately thereafter, the fluorescence spectrum was recorded in the range of 600 to 800 nm with excitation at 580 nm. The free or non-functionally bound phycobilisome fluorescence peak was observed at 625 nm. The fluorescence spectra were deconvoluted and quantified in Origin software (OriginLab Corporation, Northampton, MA, USA). Phycobilisomes fluorescence was expressed as a percentage of the total fluorescence of all components, i.e., PBS, PSII, and PSI.

**Figure 5:**
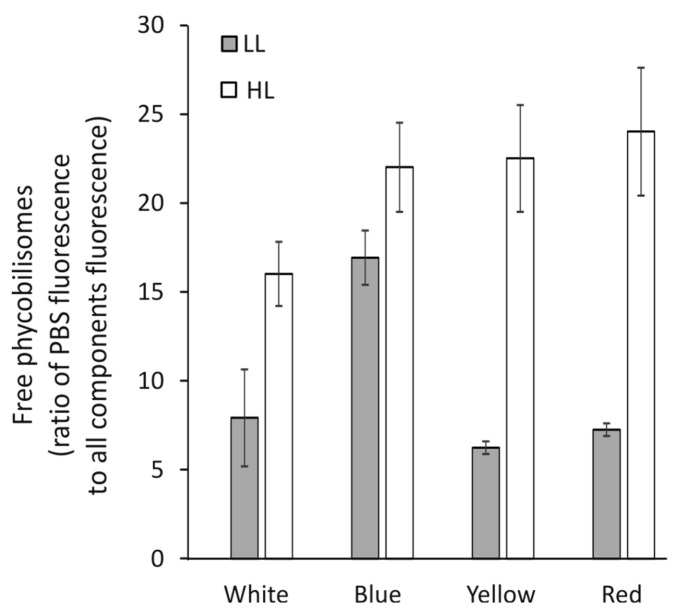
The ratio of PBS fluorescence to total fluorescence (PBS, PSII, and PSI) in *C. merolae* cells measured at 77 K, expressed as a fraction of the area of peak λ = 625 nm to total fluorescence of *C. merolae* cells growing in white, blue, yellow, and red light. The low (LL) and high (HL) intensity of growth light is marked with gray and white bars, respectively.

It was observed (Figure 5) that in high light intensity, during cell growth in all light qualities, the level of free phycobilisomes increased compared to low light conditions. The fluorescence ratio of the cells grown only under low blue light was two times higher than the control conditions under white light, which indicates double amounts of free phycobilisomes. Other light qualities (Y and R) showed a ratio of the free phycobilisomes similar to those of the white control. The high light intensity of all growth light colors increased the number of free phycobilisomes by ≈20% as compared to white light. In the growth conditions of Y and R-HL, the level of free phycobilisomes increased about three times compared to low light.

### 3.6. Induction of Phycobilisome Detachment by Short Exposure to High Light Intensity

**Figure 6:**
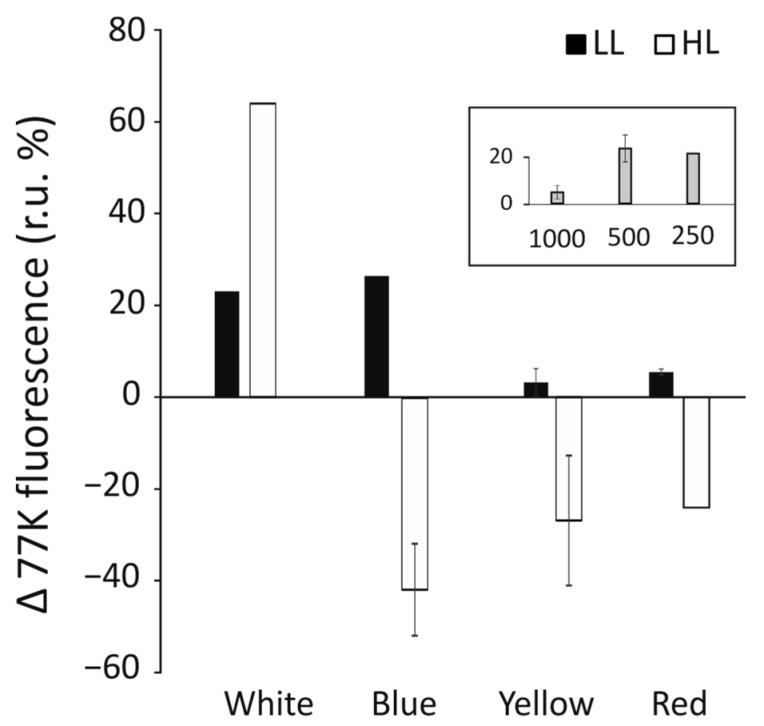
Changes in the relative rate of fluorescence intensity (in %) of free phycobilisomes after light pulses of 500 µmol photons m^−2^ s^−1^ of *C. merolae* cells growing in white, blue, yellow, and red light. The low (LL) and high (HL) intensity of growth light are marked with black and white bars, respectively. Insert: Changes in fluorescence intensity of white low-light grown cells upon light pulses of three selected intensities (250, 500, and 1000 µmol photons m^−2^ s^−1^).

Cells from all growth conditions were brought to identical OD_720_ = 0.2, and the same sample was divided into two cuvettes. One was mixed in equal volume with 50% glycerol and snap-frozen in liquid nitrogen, while the other was first exposed to 30 s of 500 µmol photons m^−2^ s^−1^ irradiation with white light, subsequently mixed with 50% glycerol, and snap-frozen in liquid nitrogen. The fluorescence spectra were acquired in the range of 600 to 800 nm, with a 580 nm excitation wave. Cells grown in W-LL and B-LL were observed to increase their free phycobilisomes fluorescence (λ = 625 nm) peak by ≈20–30%, while for cells grown in yellow and red light, the increases were only by 10–15% (Figure 6).

It was observed that in HL grown cells, only white light growth conditions allowed for a significant increase of ≈65% in the fluorescence of free phycobilisomes, while cells from the remaining HL growth conditions exhibited a 30–40% reduction in the fluorescence of free phycobilisomes, possibly due to photoinhibition. The inset of Figure 6 presents changes in the PBS fluorescence of cells grown in low white light and irradiated by various (250–1000 µmol photons m^−2^ s^−1^) light intensities. The pulse of 500 µmol photons m^−2^ s^−1^ caused the highest level of dissociation of phycobilisomes. Higher light intensity probably caused significant damage to the ability of the phycobilisomes to detach or to emit fluorescence.

### 3.7. Cell Energization, and ATP and ADP Concentration

**Figure 7:**
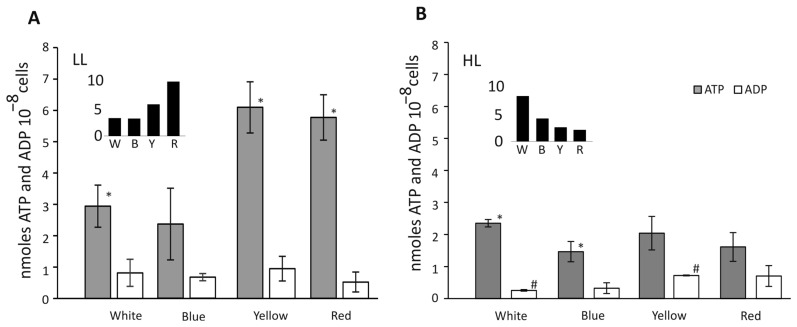
Concentration of ATP and ADP in *C. merolae* cells grown in low light ((**A**), 25 μmol photons m^−2^ s^−1^) and high ((**B**), 100 μmol photons m^−2^ s^−1^) (W), blue (B), yellow (Y), and red (R). The amounts of ATP and ADP are marked with gray and white bars, respectively. The insets show the ATP/ADP ratios. The *t*-test (T.TEST, Excel, Microsoft) returned statistically significant results (*p* < 0.05) marked as * for ATP and # for ADP.

Cells grown under all the defined experimental conditions were counted in the Neubauer chamber (VWR, Germany), and an equal number of cells under the above conditions (5 × 10^8^ cells) were harvested for analysis of ATP and ADP. It was observed that the concentration of ATP was highly dependent on growth light conditions and was much more variable in low light (Figure 7).

ATP and ADP levels were lower under HL than in LL in all light growth conditions. The control conditions (white light) exhibited only a ≈15% decrease in ATP in the high light of B-HL and remained similar in Y-HL and R-HL. The ADP levels in HL remained similar in B-HL and double in Y-HL and R-HL. B-LL reduced the amount of ATP and ADP by ≈15%, but the change was not statistically significant. The greatest difference was observed in Y-LL and R-LL, where it caused the concentration of ATP to double with almost unchanged levels of ADP compared with the white light (Figure 7).

The ATP/ADP ratio (inset) differed between light growth conditions and was highest for Y-LL and R-LL (6.3 and 10.9, respectively). For cells grown in HL, the highest level of energization was observed in W-HL. B-HL caused it to drop by 50%, with the most significant decrease in Y-HL and R-HL (2.5 and 2.3, respectively).

### 3.8. Starch Accumulation in C. merolae Cells

**Figure 8:**
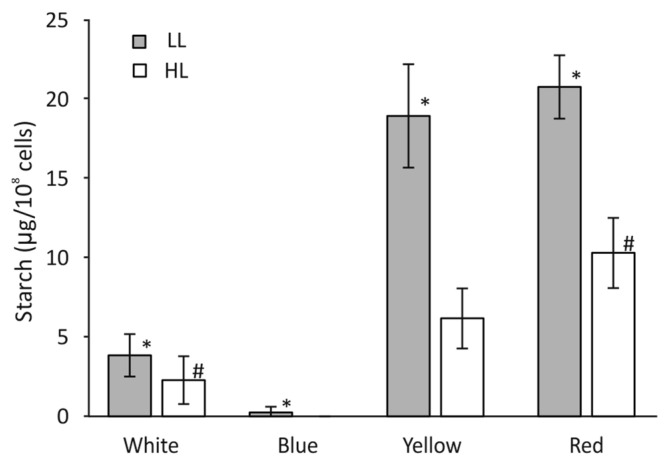
Starch accumulation in *C. merolae* cells grown in W, B, Y, and R light at LL (gray bars) and HL (white bars) intensity. The *t*-test (T.TEST, Excel, Microsoft) returned statistically significant results (*p* < 0.05) marked * for LL and # for HL.

A volume containing 50 × 10^8^ cells from each growth condition was used for starch concentration measurements. It was observed that starch accumulation was ≈30% higher in low-intensity white light than in high light (Figure 8). Both the B-HL and B-LL light conditions abolished any accumulation of starch. In contrast to B, both Y-LL and R-LL allowed for a very high level of starch accumulation. Starch concentration was more than four times the control level under Y-LL conditions and five under R-LL conditions. The Y-HL and R-HL conditions also induced starch production; however, this was only a third of its Y-LL level in Y-HL and half its R-LL level in R-HL. The accumulation in Y and R light was correlated with higher photosynthetic activity under these conditions.

## 4. Discussion

Red algae contain two types of light-harvesting antenna systems, namely, phycobilisomes and chlorophyll *a*-binding polypeptides. In this study, *C. merolae* cells were grown in various light intensities and qualities. The structural and functional properties of the cells were investigated. The growth rates of *C. merolae* under white light were similar to those reported previously [23]. The growth rates were calculated from the angulation of the linear part of the growth curves, where the apparent growth was the fastest. For most photosynthetic organisms, the exponential phase of growth may assume a more linear shape because of self-shadowing effects. For *C. merolae,* initial growth may indeed be exponential, but the observable (recorded) phase appeared to be linear (Figure 1). The late phase of growth (not presented) appeared to plateau, probably due to excessive self-shadowing effects, accumulation of toxins in the medium, or depletion of available microelements. White light induced the fastest growth at 100 µmol photons m^−2^ s^−1^ and appeared to be a highly reproducible effect, which was also reported by another group [10]. Any higher light intensity (250 µmol photons m^−2^ s^−1^) induced a reduction in growth rates, most likely due to photoinhibition and accumulation of damage caused by light (Figure 1 and Figure S1) [24,25]. Therefore, light greater than 100 µmol photons m^−2^ s^−1^ was rejected as it could introduce a reaction to photodamage rather than remodeling the light harvesting system itself. The selected light intensities were 25 µmol photons m^−2^ s^−1^ as the low light (LL) and 100 µmol photons m^−2^ s^−1^ as the high light (HL). Growth rates in any monochromatic light of identical intensity were slower by two to three times compared to the white control light of similar intensity. Monochromatic blue light had the strongest inhibitory effect on cell growth, possibly due to the induction of energy-quenching mechanisms, initiated by the internal conversion of the second excited state in chlorophyll molecules of both photosystems [26]. Changes in light intensity and wavelength might also be detected by photoreceptors, which subsequently induce specific physiological responses by triggering signal transduction cascades. Signaling pathways that involve the function of algal photoreceptors have been broadly investigated in vivo in a unicellular green alga, *Chlamydomonas reinhardtii*. The development of techniques that allow the light-dependent control of signaling pathways in other algae is underway [27,28]. Algae have several types of blue-light receptors that regulate different aspects of growth and development, as well as metabolic responses [29], including cryptochromes and phototropin [30]. In plants, cryptochromes are involved in light-dependent gene expression; the light dependence mainly affects genes involved in the response to biotic/abiotic stress and regulation of photosynthesis. Specifically, in higher plants, the presence of cryptochromes has been associated with seedling development, mediating blue light dependent inhibition of hypocotyl elongation, chlorophyll and anthocyanin production, and general transcription reorganization [31]. Algae are rich in multiple phytochromes and proteins of the photolyase/cryptochrome family, covering the entire visible spectral region [32,33]. Therefore, it can be postulated that light of a specific wavelength can induce physiological and anatomical transformations that affect metabolic processes *through* specific cryptochromes; for example, blue light in *C. reinhardtii* induces the formation of gametes [34]. Cryptochromes, unlike photolyase, do not possess a DNA repair function. Since no photolyase activity was observed in the CRY-DASH class of cryptochrome/photolyase enzymes using in vitro and in vivo photolyase complementation assays, these enzymes were initially labeled cryptochromes [25]. The CRY-DASH proteins, also known as ssDNA photolyases, were discovered to be photolyases with high specificity for CPDs (repair cyclobutene pyrimidine dimer) in single-stranded DNA in later research [33]. CRY-DASH (*Drosophila, Arabidopsis, Synechocystis, Homo*)-type proteins, a separate subfamily of cryptochromes [35,36], identified in organisms from bacteria to vertebrates [37] appear to perform a repair activity of single- and double-stranded DNA. *C. merolae* has three genes similar to CRY-DASH:CYME_CMA044C on chromosome 1, CYME_CMJ130C on chromosome 10, and CYME_CML116C on chromosome 12 [25], and we propose that activation of any or all of these genes may lead to decreased growth under blue light conditions. More research is needed, especially with CRY-DASH K.O. mutants, that could envisage the role of these proteins more clearly.

Organelle divisions and the *C. merolae* cell cycle, which divides synchronously with the light/dark cycle, are closely related processes. These cyclical processes are necessary for the division, development, and differentiation of this photosynthetic red alga, probably regulated by a circadian clock, and are induced by photoreceptors [38]. Furthermore, the regulation of the photosynthetic machinery in *C. merolae* for both microtubule-independent and light-synchronized cells depends on the transduction of the light signal [39]. It is also possible that the alga uses a less complex regulating mechanism that does not rely on a circadian clock but rather on light sensing and signal transduction. The latest studies have shown that CRY regulates growth and development in plants and the circadian clock in animals [40].

Physiological changes observed in cells grown in B-HL and B-LL seem to suggest that together with the abrogation of starch (Figure 8), the abundance of PSII proteins has been upregulated 2.5 to 3 times (Figure 2), and the level of respiration was increased threefold as compared with the white light control (Figure 3D). These results might explain the lowest growth cell rate under blue light [41]. The overexpression of PSII components could act as a cellular means for increasing linear electron transfer. Blue light highly induces respiration, dependent on light intensity during growth, about five times and two times higher, in low and high light, respectively, as compared to white light (Figure 3D). This suggests that the accumulated products of photosynthesis are constantly being used in the Krebs cycle. However, the content of ATP was similar in both blue light intensities as in the white light of both intensities (Figure 7). In B-LL intensity, the ATP/ADP ratio was 3.5 that of the white control, while in B-HL, the ratio was 4.5, which was two times lower than in the white control (9.0), suggesting diminished but still sufficient levels of cell energization. Moreover, the initial rates of oxygen evolution appeared to be similar to those of the white control (Figure 3A). Possibly, the blue-light-grown cells use up much more of their metabolic resources on maintaining the three times higher levels of PSII molecules to yield just half of the output as in white light. This could be caused by the fact that similar amounts of phycocyanin (R-PC) antennas are produced in B-LL and B-HL (Figure 2D) but nearly twice as many are not functionally bound to any photosystem (Figure 5). It appears that blue light simultaneously triggers protective reactions such as dissociation or uncoupling of phycobilisomes and photosynthetic starvation reactions such as overproduction of PSII and R-PC antennae.

Yellow and red light produce very different assemblies of the photosynthetic apparatus, being optimized differently. Both red and yellow light induce very similar growth rates in all investigated light intensities (Figure 1). The concentration of PSII components was about 50% lower than in the white-light control in both light intensities (the high red light induced a level similar to that in high white light). Both Y-LL and R-LL reduced the cellular abundance of R-PC by 60% (Figure 2D), while simultaneously, similar amounts of R-PC remained unbound or unfunctional (Figure 5). Importantly, the bound fraction of PBS appeared to bind more strongly with photosystems and was not easily detached upon high light pulses (Figure 6). Because yellow and to some extent red light is capable of being absorbed by phycobilisomes, cells grown in these light conditions would undergo optimization of their photosynthetic apparatus. These changes cause a reduction in PSII molecules with phycobilisomes of larger APC cores but shorter R-PC rods as main light harvesters. Interestingly, the HL conditions seemed only to exaggerate the changes in the shape of phycobilisomes by further increasing the contribution of the core proteins (Figure 2C). The reduction in rod length appeared to be related to a larger free PBS fraction (Figure 5), rather than to decreased expression levels (Figure 2D).

Cells grown in Y-LL and R-LL appeared to generate twice as high an energization level (Figure 7) and three times the level of starch accumulation (Figure 8) compared to the W-LL control, suggesting an oversupply of primary products of photosynthesis. The high intensity of yellow and red light decreased the ATP levels to that of high white light control, together with starch production, but still kept it at significant (*p* < 0.05; Figure 8) and three times higher levels than the white control. Our results may suggest that the quality and intensity of the growth light played a role in supplementing ATP, which was needed to drive cell metabolism under different light conditions. A range of regulatory system(s) come into play that result in modulating ATP production and maintaining energy homeostasis. Electrochemical gradients of protons are known to drive ATP synthesis and respond to changes in the physiological and metabolic states of the cell [42].

There was a significant difference between yellow and red light in the amount of chlorophyll *a* found in the respective cells (Figure 4). The yellow light cells contained only 25% of the chlorophyll *a* of the white control, while in red light, the value was between 50% and 60%. That suggests that red-light-grown cells still utilize Lhcr antennae for light harvesting, while yellow-light-grown cells probably harvest much more of their energy via phycobilisomes. That is consistent with the light absorption properties of chlorophyll *a* as phycobilisomes exhibit their absorption maxima in red and yellow light, respectively. Chlorophyll *a* concentration was lower than previously reported [43] for the larger (8–14 µm) *Pseudokirchneriella subcapitata* and ranged between 60 and 220 nmol per 10^8^ cells. It was larger than similarly sized (≈2 µm) *Synechocystis* sp. PCC 6803 [44] at a maximum level of 4.5 nmol 10^−8^ cells.

We propose that both light quality and quantity influence the remodeling of the *C. merolae* photosynthetic apparatus. Light quality has the largest influence on PSII and R-PC expression levels, while light intensity induced the expression of APC and the dissociation of R-PC from the PBS assembly. It was proposed previously [10] that APC exhibits ineffective transfer of energy to PSII; therefore, the larger APC core might act as an effective light filter, preventing excess light from being transferred to PSII reaction centers. Possibly, the 3–4 times higher level of free R-PC in high-light-grown cells (Figure 5) also acts as a photoinhibition protector by absorbing excess light and reemitting it in a random direction, effectively shielding the photosystems. In this way, it could act as a light-scattering agent [45].

The highest level of functionally bound PBS was observed in low blue light (Figure 3B). Under this condition, the yellow light was highly efficiently absorbed by PBS functionally bound with PSII, leading to a very rapid increase in oxygen evolution, associated with increased respiration (Figure 3B). The cells adapted to low blue light were possibly most receptive to yellow low light, and quickly achieved their maximal efficiency. The similar amount of cellular chlorophyll *a* as in the control (low white light) suggests that low blue light does not induce an increase in Lhcr antennae in PSI (Figure 2 and Figure 4) as the number of PSI core molecules remained unchanged (Figure 2B). In this condition, PBS may be bound with PSI more effectively, which, however, requires more specific investigation.

An opposite configuration of PBS, which exhibited the lowest degree of bounding to PSII, was assembled under conditions of high red and yellow growth light. Low light conditions resulted in a reduction in the total level of R-PC (Figure 2D), coupled with a similar level of free PBS in the cells (Figure 5) similar to that of the white control. However, high-light conditions yielded similar levels of R-PC (Figure 2D) but much higher levels of free PBS (Figure 5). Measurements of the initial photosynthesis rates in yellow light (Figure 3B) showed a very slow increase in oxygen evolution. Surprisingly, this level of photosynthetic activity was enough to support a growth rate 60% lower than in high white light (Figure 1) and a threefold higher accumulation of starch (Figure 8).

The cells showed a very different adaptation mechanism. They produced half the amount of PSII as in the white-light control (Figure 2A) and had only 25% of the total chlorophyll *a* content of the control in both light intensities. There was a remarkably similar initial activity rate in white and yellow light (Figure 3A,B, respectively) of both light intensities; simultaneously, these cells showed a negligible initial activity rate in red and blue light (Figure 3C,D, respectively), that is, the one mediated by chlorophyll *a*. This demonstrated how effectively PBS harvests yellow light, especially at low intensities. High intensities of yellow light cause the dissociation of PBS from photosystems and a significant drop in the efficiency of the photosynthetic process.

Our results show that the intensity and quality of light can differentially regulate the function of PBS, which in turn influences the physiological status of the cells in terms of the rate of growth, the intensity of O_2_ exchange, the energization level, the amount of pigments, etc. Interestingly, acclimation to particular light changes is probably realized very quickly by changes in PBS by binding/dissociation from photosystem(s) (PBS, which is weakly coupled with PS), which rapidly influences the rate of photosynthesis, as suggested by pulse-induced detachment of PBS. The importance of the light-induced adjustment of photosynthetic complexes on the mobility of PBS remains an open question that should be investigated in future experiments. Another aspect worth considering is the influence of free PBS mobility in the chloroplast. A relatively high density of membrane packing in red algae may diminish the ability of free PBS to disperse around the chloroplast and force them to associate/dissociate more locally. It was previously assessed [46] that PBSs in *C. merolae* are only moderately mobile. Therefore, we suggest that state transitions have an important regulatory role in mesophilic red algae, but in thermophilic red algae, this process was replaced by nonphotochemical quenching. The contribution of NPQ in various growth light conditions is currently being investigated in *C. merolae*.

## 5. Conclusions

Our findings revealed that the *C. merolae* cell response to light can be modulated in two ways. The first occurs in response to light quality and involves the de novo synthesis of a D1 protein and phycocyanin, while the second occurs in response to light intensity and involves the synthesis of allophycocyanin and ATP, both of which can regulate the function of PBS, which in turn influences the physiological status of cells: rate of growth, intensity of O_2_ exchange, energization level, pigment amount, etc. Importantly, variations in PBS due to binding/dissociation with the photosystem(s) (typically, PBS is weekly associated with PS) likely result in very rapid adaptation to light fluctuations, which rapidly alters the rate of photosynthesis.

Because light is the most important environmental factor, photoreceptors should indeed be involved in the regulation of *C. merolae* metabolism and behavior under changing light conditions. Data on algal light-dependent signaling are scarce, and it is possible that it is species specific.

It is also plausible that the changes in the amounts of PSII, APC, and R-PC proteins reflect changes in the structure of PBS, with larger APC cores but shorter R-PC rods. The role and contribution of NPQ in the mobility of PBS remain unknown, as does the impact of light-induced modification of photosynthetic complexes, which should be investigated in subsequent experiments.

## Supplementary Materials

The following supporting information can be downloaded at https://www.mdpi.com/article/10.3390/cells12111480/s1, Figure S1: Growth curves of *C. merolae* cells; Table S1: Primers used for generation of standard proteins (genes: *psaA*, *cpcA*, and *cpcA*).
