# Supplementary material for: How Light Modulates the Growth of Cyanidioschyzon merolae Cells by Changing the Function of Phycobilisomes
Source: Cells. 2023 May 26;12(11):1480. doi: 10.3390/cells12111480 (PMC10252272; doi:10.3390/cells12111480)
Supplement: Supplementary file 1 [file cells-12-01480-s001.zip › cells-2358170-supplementary/Supplementary Figure S1.pdf]

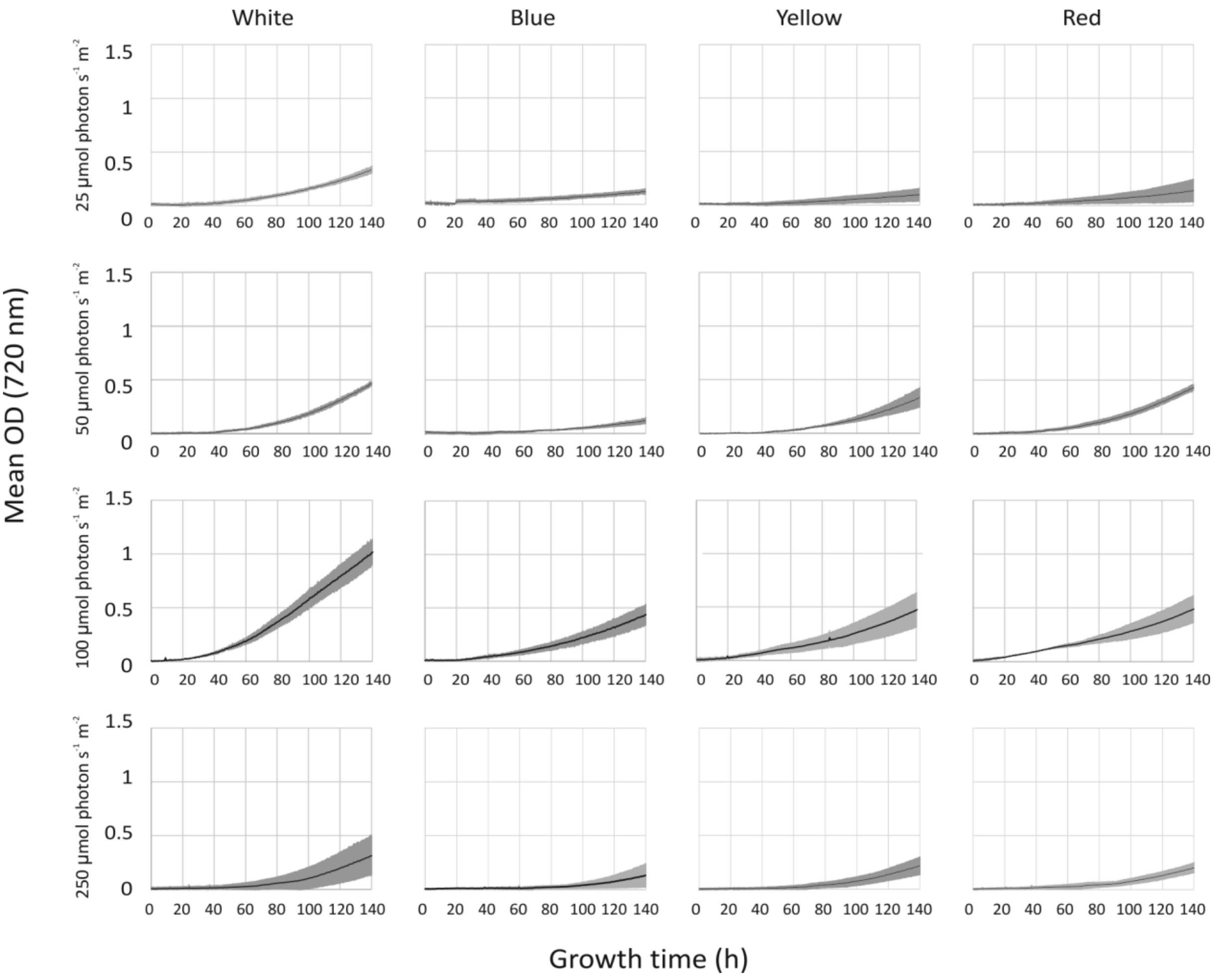

Supplementary Figure S1. Growth curves of *C. merolae* cells. OD was acquired at λ = 680 nm and 30 min intervals. The initial OD was 0.01 and that culture was carried for 7 days, out of which 140 hours were presented. Grey area represents standard deviation of the mean value (black line), calculated from at least three sets of acquired curves. Four light intensities were applied (25, 50, 100 and 250 µmol photon m^{-2} s^{-1}) in combination with three monochromatic LED sources (blue, yellow and red) and white control.
