# Supplementary material for: How Light Modulates the Growth of Cyanidioschyzon merolae Cells by Changing the Function of Phycobilisomes
Source: Cells. 2023 May 26;12(11):1480. doi: 10.3390/cells12111480 (PMC10252272; doi:10.3390/cells12111480)
Supplement: Supplementary file 1 [file cells-12-01480-s001.zip › cells-2358170-supplementary/Supplementary Table S1.pdf]

Supplementary Table S1 Primers used for generation of standard proteins (PsaA, CpcA, and CpcA).

PsaA_xho_f	ATCTCGAGATGACGTTAACAACAGAGAAACAAG
PsaA_xho_r	TGCTCGAGGCTACCGCTTCCACTTCCGCTACCTACTGCAATAATTCTTGCTAAGA
CpcA_nco_f	ATACCATGGGTAAAACTCCAATAACTGAAGCAATA
CpcA_sal_r	GTGCTCGAGCGCTTTGGCAGCCGCTTCCTTTGCGGCAGCCTCTTTAGCCGCTGCTTCGGCGCTTAATGCGTTGATAGCATA
Apc_xho_f	ATACCATGGGTATTGTTACCAAGTCAATTGTGAAT
ApcA_xho_r	GTGCTCGAGCGCTTTGGCAGCCGCTTCCTTTGCGGCAGCCTCTTTAGCCGCTGCTTCGGCTTGCATTGCATTGATAGTGTA
